# Epstein–Barr virus-related hemophagocytic lymphohistiocytosis complicated with coronary artery dilation and acute renal injury in a boy with a novel X-linked inhibitor of apoptosis protein (XIAP) variant: a case report

**DOI:** 10.1186/s12887-020-02359-4

**Published:** 2020-10-02

**Authors:** Ru-Yue Chen, Xiao-Zhong Li, Qiang Lin, Yun Zhu, Yun-Yan Shen, Qin-Ying Xu, Xue-Ming Zhu, Zhen-Jiang Bai, Ying Li

**Affiliations:** 1 Department of Nephrology and Immunology, Children’s Hospital of Soochow University, Suzhou, Jiangsu China; 2 Department of Pathology, Children’s Hospital of Soochow University, Suzhou, Jiangsu China; 3 Pediatric Intensive Care Unit, Children’s Hospital of Soochow University, Suzhou, Jiangsu China

**Keywords:** X-linked inhibitor of apoptosis protein (XIAP), Hemophagocytic lymphohistiocytosis (HLH), Epstein–Barr virus (EBV), Coronary artery dilatation, Acute renal injury (AKI)

## Abstract

**Background:**

X-linked lymphoproliferative disease (XLP) is a rare inherited X-linked primary immunodeficiency diseases (PID). One such disease, X-linked inhibitor of apoptosis protein (XIAP) deficiency, is characterized by Epstein–Barr virus-related hemophagocytic lymphohistiocytosis (EBV-HLH). However, EBV-HLH with coronary artery dilation and acute renal injury (AKI) in children is unusual.

**Case presentation:**

We report the case of a young boy aged 17 months with a novel XIAP variant. He was initially diagnosed with EBV-HLH based on the HLH-2004 diagnostic criteria and the condition was accompanied by coronary artery dilation and acute renal injury. The comprehensive genetic analysis of peripheral blood-derived DNA revealed a hemizygous variant of the *XIAP* gene [c.116G > C(p.G39A)], which was inherited from his mother (heterozygous condition). After combined treatment with rituximab, intravenous immunoglobulin, corticosteroids, antiviral drugs, and mycophenolate mofetil (MMF) in addition to supportive therapy, his clinical manifestations and laboratory indexes were improved. The patient achieved complete remission with MMF treatment in the 8-month follow-up.

**Conclusions:**

We report the [c.116G > C(p.G39A)] variant in the *XIAP* gene for the first time in a case of XLP-2 associated with EBV-HLH. For male patients with severe EBV-HLH, the possibility of XLP should be considered and molecular genetic testing should be used early in auxiliary diagnosis. Reports of EBV-HLH with coronary artery dilation and AKI in children are rare. In the patients with EBV-HLH, color Doppler echocardiography and urine tests should be monitored regularly. If necessary, renal biopsy can be performed to clarify the pathology. Treatment with rituximab, immunosuppressors and supportive therapy achieved a good effect, but long-term follow-up is required.

## Background

X-linked lymphoproliferative disease (XLP) is a rare inherited X-linked primary immunodeficiency disease (PID), which has two recognizable subtypes identified by the *XLP-1* and *XLP-2* gene variants. XLP-1 is caused by variants in the *SH2D1A* gene, which maps to the X-chromosome (Xq25–26) and contains four exons coding for the signaling lymphocytic activation molecule-associated protein (SAP) that is involved in immune cell activation. XLP-2 is caused by variants in the *XIAP* gene, also known as the *BIRC4* gene [1, 2]. *XIAP* is located close to *SH2D1A* on the X-chromosome and contains six exons that encode the X-linked inhibitor of apoptosis (XIAP) protein [1]. Both of these variants are associated with defective immune responses to Epstein–Barr virus (EBV) infection [3]. Here, we report the case of a young boy with cardiovascular and renal lesions who was diagnosed with EBV-positive hemophagocytic lymphohistiocytosis (EBV-HLH) secondary to an underlying *XIAP* gene variant [c.116G > C(p.G39A)].

## Case presentation

A small boy aged 17 months (body weight, 10 kg; height, 88 cm; ethnicity, Chinese) was admitted to the Infection Department of our hospital for persistent fever lasting 8 days. Neither the boy nor his family had a significant history of conditions such as cancer, inflammatory bowel disease (IBD), and autoimmune diseases. However, the mother had a history of gestational diabetes, hypothyroidism and anemia during pregnancy, which improved after 2 months of active treatment.

After 4 days of anti-infective treatment, the child still had a high fever and presented with lethargy, abdominal distention, oliguria and vomiting. The auxiliary examination suggested that multiple organs were involved and multiple serous cavity effusion was present. The child was transferred to our department and more relevant examinations were applied. He developed a high fever, polyserositis (pelvic, pleural, peritoneal and pericardial effusion), anemia (hemoglobin (Hb) 70 g/L), hyperferritinemia (1149.4 ng/ml), hypertriglyceridemia (4.65 mmol/L), obvious elevated soluble CD25 (5783.3 pg/ml; reference range: 400–2500 pg/ml), mildly increased IL-6 (10.3 pg/ml; reference range: 0–2.2 pg/ml) and IL-10 (12.8 pg/ml; reference range: 0–2.3 pg/ml). The proportion of CD3-CD56^+^ cells was 1.2% (reference range: 3.3–32.3% in lymphocyte). Killer cell immunoglobulin (Ig)-like receptor (KIR) expression and function in NK cells was normal. EBV shell and early antigen IgG antibody were positive, with low affinity of capsid antigen. High EBV DNA loads in peripheral blood and serum (6.69 × 10^4^ copies/ml and < 5.0 × 10^2^ copies/ml, respectively) suggested a diagnosis of EBV-HLH based on the HLH-2004 diagnostic criteria. Bone marrow biopsy was negative for hemophagocytes. Hemolysis tests and ADAMTS13 activity were normal. Other pathogen detection tests such as blood culture, sputum culture, T-SPOT, and mycoplasma antibody tests were negative. Color Doppler echocardiography indicated coronary artery dilatation with mild pericardial effusion and valvular regurgitation (Table 1). The progressive increase in serum creatinine and decrease in estimated glomerular filtration rate (eGFR = 9 ml/min/1.73m^2^ according to the Schwartz equation), combined with oliguria, persistent proteinuria and hematuria suggested acute kidney disease (AKI). Due to the young age of the child, it was difficult to collect 24-h urine; therefore, 24-h urine protein quantification was not performed. Comprehensive genetic analysis (full exhome sequencing) of peripheral blood-derived DNA revealed that the patient carried a hemizygous variant of XIAP (NG_ 007264.1, NM_ 001167, NP_001158.2). The maternal missense variant c.116G > C in exon 2 leads to an amino acid change (p.G39A), which has never been reported previously and is absent from controls (1000 Genomes, ExAC, gnomAD, and CNGB). SIFT program predicts that the variant is damaging (score 0.012 < 0.05), which might affect normal functions of the protein [4]. According to the standard of American College of Medical Genetics (ACMG) [5], variant c.116G > C is classified as Variant of Uncertain Significance (VUS) of XIAP. However, the patient in our study presented with typical clinical phenotypes of EBV-HLH. In addition, we didn’t have identified any other mutations that were associated with this disease of the patient. Thus, we predicted that the variant c.116G > C of XIAP should be responsible for EBV-HLH.

**Table 1:** Color Doppler echocardiography

Time	EF%	FS%	Coronary artery diameter (mm)							Hydropericardium	Cardiac valves
			LMCA	Z	LAD	Z	Proximal RCA	Z	Aortic annulus		
1d	80	50	2.2	0.91	–	–	1.7	0.22	10	–	–
8d	68	37	2.3	1.19	2.1	1.98	1.7	0.19	10	Mild	Mild regurgitation of mitral valve, tricuspid valve and aortic valve
11d	66	36	2.5	1.75	2.3	2.58	2.5	2.38	11.6	Mild	Mild regurgitation of mitral valve, tricuspid valve and aortic valve
27d	72	40	2.4	1.47	2.3	2.58	2.1	1.29	11.6	–	–
40d	70	39	2.5	1.71	2.3	2.54	2.2	1.52	10.3	–	–
78d	63	33	2.5	1.63	2.2	2.17	2.1	1.17	11.6	–	–
132d	68	37	2.4	1.27	2.0	1.51	2.1	1.1	12	–	–

*LMCA* Left main coronary artery, *Proximal RCA* Proximal right coronary artery, *LAD* Left anterior descending artery

**Fig. 1:**
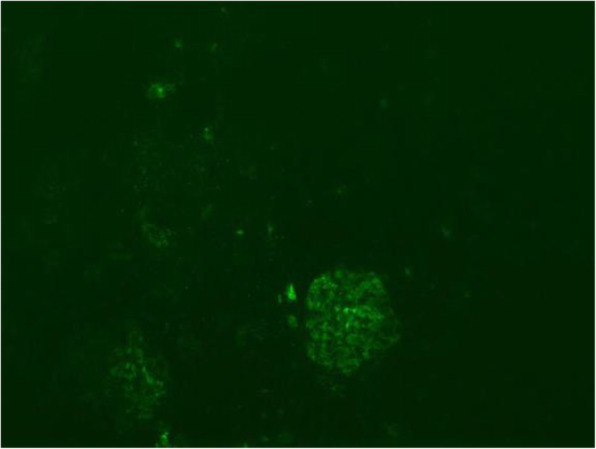
Immunofluorescence of renal biopsy (× 100). Focal deposition of immunoglobulin-M in glomerular mesangium and capillary loops

**Fig. 2:**
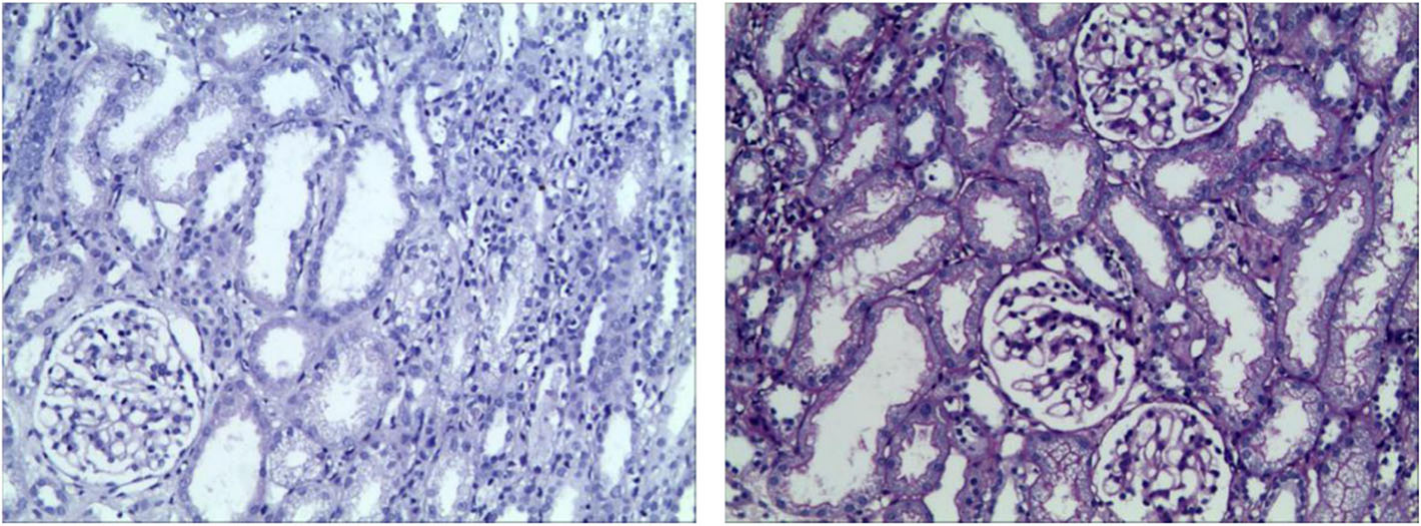
Light microscopy of renal biopsy (×100). Mild mesangial hyperplasia in glomeruli; vacuolar degeneration and atrophy of epithelial cells in some renal tubules; chronic inflammation of stroma, scattered infiltration of lymphocytes and slight hyperplasia of fibers

**Fig. 3:**
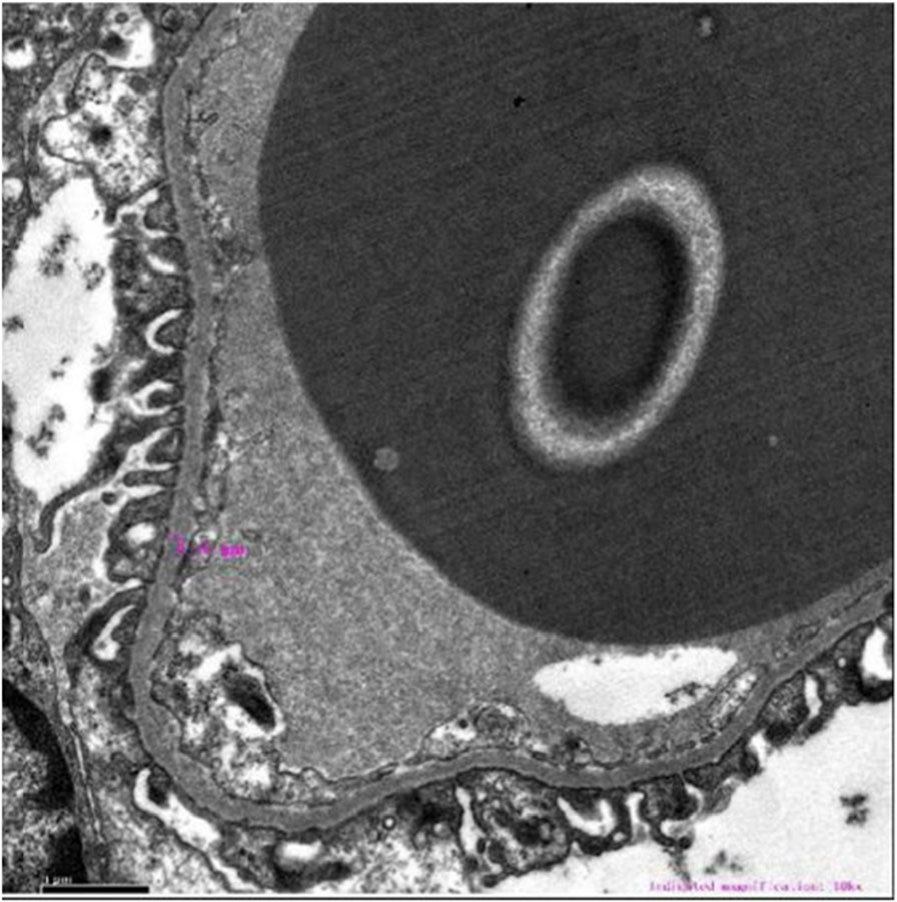
Electron microscopy of renal biopsy (× 10,000). Glomerular capillary basement membrane are thin segmentally (160–280 nm)

After 1 month of a treatment regimen including intravenous immunoglobulin (2 g/kg), methylprednisolone pulse (MP; 20 mg/kg.d × 3d, 8 mg/kg.d and then gradually reduced), antibiotics, antiviral drugs (acyclovir and ganciclovir sequentially), rituximab (0.1 g), aspirin, and urokinase in addition to supportive therapy, the patient’s clinical manifestations and laboratory indexes were obviously improved except for coronary artery dilatation, persistent proteinuria (2 + − 3+) and hematuria. Renal biopsy was then performed (Figs. 1, 2 and 3). Under light microscopy, the glomeruli showed mild mesangial hyperplasia combined with vacuolar degeneration and atrophy of epithelial cells in some renal tubules, and dilatation of the tubular lumen. Extended extracellular matrix with scattered infiltration of lymphocytes and slight fibrosis suggested chronic renal tubulointerstitial inflammation. Immunofluorescence analysis showed focal deposition of IgM in glomerular mesangium and capillary loops. Under electron microscopy, the glomerular capillary basement membrane was thin (160–280 nm), with segmental fusion of epithelial podocytes, and no electron dense deposition. The patient’s treatment was continued with mycophenolate mofetil (MMF) and 5 days later, his urine protein test was negative, although hematuria continued. After 3 months, color Doppler echocardiography showed no obvious dilation of the coronary artery. After 6 months, the hematuria was in remission and the child had a good prognosis in the regular 8 month follow-up.

## Discussion and conclusion

X-linked lymphoproliferative disease (XLP) is a rare form of X-linked PID, that occurs in two forms known as XLP-1 and XLP-2 [6]. The annual incidence of XLP is approximately 2–3 cases per million, of which XLP-1 accounts for more cases than XLP-2 [6]. In China, the onset of XLP is usually at 1–60 months of age, and is often triggered by EBV infection [1]. Other viral infections, such as human herpes virus 6, cytomegalovirus, have also been reported in association with XLP, although cases without an identifiable infectious agent have also been described [7, 8]. The life-threatening manifestations of XLP triggered by EBV include EBV-positive hemophagocytic lymphohistiocytosis (EBV-HLH), chronic active EBV infection (CAEBV) and EBV-positive lymphoma [3].

### Clinical manifestation

XLP-1 and XLP-2 patients differ in terms of several clinical features. Patients with XLP-1 develop recurrent splenomegaly and IBD very rarely, while patients with XLP-2 do not develop lymphoma. Hypogammaglobulinemia is common to XLP-1 and XLP-2 [7]. HLH is common in XLP-2, and often recurrent [2, 6, 9]. According to an international survey, approximately 55% of patients with XLP-2 developed HLH and 26% presented with IBD, which was lower that the HLH frequency in Japan (79%), France (71%) and the USA (82%) [7, 10]. In a genetic study of 265 Chinese patients with HLH, genetic variants were observed in 87 (32.83%) patients, of which 10 (11.49%) had variants in *XLAP* and six (6.90%) carried variants in *SH2D1A* [11]. EBV infection has been reported to be a trigger of the first HLH episode in patients with XIAP deficiency (30–70% of cases) [6, 8, 9]. The comprehensive genetic analysis of peripheral blood-derived DNA in our case revealed a hemizygous variant of the *XIAP* gene [c.116G > C(p.G39A)], which was inherited from the patient’s mother (heterozygous condition). The patient was diagnosed with EBV-HLH based on the HLH-2004 diagnostic criteria according to clinical manifestations and relevant examinations, which was consistent with the characteristics of the *XIAP* gene variant. Family history provides critical information for the identification of XLP; however, the child in this case had no family history of IBD or lymphomas.

### Cardiovascular complications

In this case, the boy developed recurrent fever and relevant examinations suggested the diagnosis of EBV-HLH with coronary artery lesions similar to Kawasaki disease (KD). However, the patient lacked the typical symptoms of KD, such as skin rash, conjunctivitis, swelling or desquamation of the extremities. As stated previously, both KD and EBV-HLH are autoinflammatory diseases complicated by immunologic dysfunction. There is considerable overlap between these conditions in terms of pathophysiology, such as increased serum levels of IL-6, IL-8, TNF-α and soluble IL-2 receptor [12]. Some studies indicated that EBV-infected CD8^+^ T cells and high levels of the cytokines in EBV-HLH may play some role in development of coronary artery lesions [12, 13]. Recent studies have shown that the serum levels of soluble CD163 and the soluble tumor necrosis factor receptor II/I ratio were significantly elevated in patients with EBV-HLH compared to those in patients with acute-phase KD [14, 15]. At present, reports of EBV-HLH with cardiovascular complications in children are rare although the cases of two Japanese girls, aged 3- and 4-years have been published. In terms of cardiovascular complications, the 3-year-old girl presented as left main coronary artery dilatation, which improved after treatment with cyclosporine, dexamethasone and etoposide [12], while the 4-year-old presented with acute myocarditis and coronary aneurysms, which improved after rituximab treatment [13]. Both children had good prognosis after long-term follow-up. See BT et al. reported the case of an 8-year-old girl from Malaysia presented with EBV-HLH associated with a giant aneurysm and ectasia of the coronary arteries [16]. The coronary lesions improved after 6 months of treatment with dexamethasone and etoposide. Sun G et al. reported a 10-year-old Chinese girl who was diagnosed with EBV-HLH with cardiac complications, including pericardial effusion (PE) and coronary artery aneurysms (CAAs) [17]. After 2 weeks, ultrasonic cardiograms revealed that the PE disappeared and no marked changes in the CAAs following treatment with dexamethasone, etoposide, aspirin and persantin. After 10 months, the PE did not reoccur and there were no obvious changes in the CAAs. However, CAEBV with cardiovascular complications has been reported in a relatively large number of patients, with the coronary artery disease being the most common complication [18–20].

### Renal lesions

In our case, the patient’s condition was manifested as oliguria, persistent proteinuria and hematuria. The progressive increase in serum creatinine level and decreased eGFR suggested acute kidney disease (AKI), which in this case may be accounted for as follows: (1) Severe storms of inflammatory cytokines result in polyserositis and hypovolemia, which cause renal hypoperfusion. Aulagnon F et al. [21] reported that the main causes of AKI during HLH were acute tubular necrosis (49%), hypoperfusion (46%), tumor lysis syndrome (29%), or HLH-associated glomerulopathies (17%). It has been reported that the leading causes of EBV-related AKI include rhabdomyolysis and hepatic failure [22]. The prerenal factors can lead to AKI. (2). Application of nephrotoxic drugs preceding treatments such as antibiotics can cause renal injury [23]. (3) Hyperactivated cytotoxic T-lymphocytes and macrophages cause direct damage to kidney tissue. Ozgurhan G et al. reported that the renal biopsy in a child with EBV infection showed intense and mixed tubulointerstitial inflammatory infiltration that was rich with T cells and histiocytes [24]. The renal biopsy in our case showed mild mesangial hyperplasia of the glomeruli, lymphocyte infiltration and mild fibrosis of the renal interstitium under light microscopy. Suzuki J et al. reported an adult case of fulminant EBV infection with AKI. In situ hybridization of EBV-encoded RNA 1 did not show the presence of the virus in the kidney and AKI was thought to be caused by cytokine secretion from activated cytotoxic (CD8^+^) T lymphocytes [23]. Moretti M et al. systematically reviewed 38 patients with primary EBV infectious mononucleosis complicated by AKI, including 27 cases of acute interstitial nephritides (AIN), three of hemolytic uremic syndromes, one of jaundice-associated nephropathy and seven of myositides [25]. Mansur A et al. [26] performed immunohistochemical staining of renal tissue from 78 patients with EBV infection and AIN and identified a positive correlation between CD68 macrophage infiltration and serum creatinine concentration, as well as expression of IL-4, eotaxin, CCR3, CCR5 and VCAM-1 in biopsies from patients with AIN. In addition, EBV was not detected by in situ hybridization and immunohistochemistry analyses in any of the AIN sections, indicating that EBV is not a pathogenetic factor in AIN [26]. In addition to AIN, EBV-related renal lesions include glomerular abnormalities such as immune-complex mediated glomerulonephritis, membranous nephropathy, minimal change nephritic syndrome and IgA nephropathy, which are very rare [27–29]. Renal biopsy immunofluorescence analysis in our case showed focal IgM deposition in the glomerular mesangium and capillary loops. (4) In situ hybridization analysis has revealed direct EBV infection in renal proximal tubular cells in patients with idiopathic chronic tubulointerstitial nephritis [30], indicating that EBV directly damages renal tissue.

### Treatment and prognosis

In cases of XLP-related EBV-HLH, EBV-infected cells are predominantly B cells [3]. Therefore, in our case, combination therapy of rituximab and ganciclovir was administered, which cleared the EBV DNA titers and relieved symptoms. It has been reported that immunosuppressive medication such as corticosteroids are not effective against this virus-associated tubulointerstitial nephritis [23]. In our case, serum creatinine and eGFR returned to normal, urine protein tests became negative, and hematuria was improved after treatment with corticosteroids and MMF. Renal replacement therapy can be given in the acute stage of renal injury. It has been reported that 47% of patients with EBV infection and AKI required renal replacement therapy, although 90% can be relieved [25]. However, it has also been reported that the incidence of AKI during HLH is 62%, with 59% cases requiring renal replacement therapy and chronic kidney disease in 32% at 6 months [21]. Other treatments for XLP include antiviral agents, anti-IFN-γ and α, intravenous immunoglobulins, etoposide, and T cell immunosuppression, but the only curative therapy is allogeneic hematopoietic stem cell transplantation (HSCT) [1, 8, 10]. It is recommended that HSCT should be performed as soon as possible for patients with EBV-HLH, hypogammaglobulinemia, lymphoma or aplastic anemia, because the outcome without HSCT is extremely poor [1]. However, the child in our case had stable vital signs, and his laboratory indexes and color Doppler echocardiography trend gradually returned to normal after the treatment of intravenous immunoglobulin, methylprednisolone, antibiotics, antiviral drugs, rituximab, aspirin, and urokinase in addition to supportive therapy, without HSCT. It is necessary to him closely in the long-term in case of disease progression or recurrence.

We report the case of a 17-month-old boy diagnosed with EBV-HLH complicated by cardiac and renal lesions. Reports of EBV-HLH with coronary artery and renal complications in children are relatively rare. Color Doppler echocardiography and urine tests were monitored regularly. Renal biopsy was performed to clarify the pathology. The application of rituximab, immunosuppressants and corticosteroids combined antiviral drugs had a good effect. The genetic analysis determined a novel hemizygous variant of the *XIAP* gene [c.116G > C(p.G39A)]. For male patients with severe EBV-HLH, the possibility of XLP should be considered and molecular genetic testing should be performed early in the auxiliary diagnosis.

## Data Availability

Data were collected from Children’s Hospital of Soochow University, Guangzhou jinyu Medical Laboratory Center, Beijing jinzhun Gene Technology company and Jiajian Check Medical Testing Corporation. These data are reliable and available.

## Abbreviations

XIAP: X-linked inhibitor of apoptosis protein
XLP: X-linked lymphoproliferative disease
PID: Primary immunodeficiency diseases
EBV-HLH: Epstein–Barr virus-positive hemophagocytic lymphohistiocytosis
EBV: Epstein–Barr virus
HLH: Hemophagocytic lymphohistiocytosis
AKI: Acute kidney disease
MMF: Mycophenolate Mofetil
SAP: Signaling lymphocytic activation molecule-associated protein
BIRC4: Baculoviral IAP repeat-containing protein 4
IBD: Inflammatory bowel disease
KIR: Killer cell immunoglobulin (Ig)-like receptor
ACMG: American College of Medical Genetics
VUS: Variant of Uncertain Significance
MP: Methylprednisolone pulse
CAEBV: Chronic active EBV infection
KD: Kawasaki disease
PE: Pericardial effusion
CAAs: Coronary artery aneurysms
AIN: Acute interstitial nephritides
HSCT: Hematopoietic stem cell transplantation
